# Odorant-binding proteins expression patterns in recently diverged species of *Anastrepha* fruit flies

**DOI:** 10.1038/s41598-017-02371-2

**Published:** 2017-05-19

**Authors:** Emeline Boni Campanini, Carlos Congrains, Felipe Rafael Torres, Reinaldo Alves de Brito

**Affiliations:** Departamento de Genética e Evolução, Universidade Federal de São Carlos, São Carlos, Brazil

## Abstract

We studied two species of closely related South American fruit flies, *Anastrepha fraterculus* and *Anastrepha obliqua* which, despite being able to interbreed, still show some ecological and reproductive differences. Because part of these differences, such as host and mate preferences, may be related to olfactory perception, we focused our investigation on the differential expression of Odorant-binding protein (OBP) gene family, which participate in initial steps of the olfactory signal transduction cascade. We investigated patterns of expression of eight OBP genes by qPCR in male and female head tissues of both species. The expression patterns of these OBPs suggest that some OBP genes are more likely involved with the location of food resources, while others seem to be associated with mate and pheromone perception. Furthermore, the expression patterns obtained at different reproductive stages indicate that OBP expression levels changed significantly after mating in males and females of both species. All eight OBP genes analyzed here showed significant levels of differential expression between *A*. *fraterculus* and *A*. *obliqua*, suggesting that they may hold important roles in their olfactory perception differences, and consequently, may potentially be involved in their differentiation.

## Introduction

Fruit flies of the *Anastrepha fraterculus* group (Diptera: Tephritidae) are some of the most important fruit pests in South America, and exhibit a limited number of morphological and genetic distinguishing characters^1, 2^, possibly because they have diverged recently^3^. Furthermore, interspecific viable hybrids can be obtained in laboratory for some of the crosses between species in the group^2, 4^, suggesting that reproductive isolation is still incipient and incomplete across the genome, which makes this group an interesting model to study the genetic and evolutionary processes involved in speciation events, since very few genes would have had the time to differentiate. Even though there is still limited information on speciation processes affecting species of this group, a lot has been learned from investigating other tephritids, such as the medfly *Ceratitis capitata* ^5^ and species of the genus *Bactrocera* ^6–8^ and *Rhagoletis* ^9^. For instance, genes involved with host race formation and olfactory reception have been shown to have important roles in the differentiation of *Rhagoletis pomonella* ^9^. Such genes may impact speciation because olfactory responses control social and sexual interactions between individuals of the same species, through the detection of odors and pheromones essential for survival and reproduction^10, 11^.

The initial steps of the transduction cascade of olfactory signals in insects, mediated by the Odorant-binding proteins (OBPs), are the solubilization and transport of chemical signals through the aqueous lymph of sensillas to reach the olfactory receptors^12, 13^. Different OBPs have specific affinities to odorants and their high molecular divergence, as well as the distinct expression patterns reported to OBP genes in insects, suggest that these proteins could act as a filter, selecting the odorants to trigger olfactory responses^14^. Although not all odorants are necessarily associated with an OBP to stimulate olfactory receptors^15^, it has been suggested that a combination of the number and types of OBPs expressed in a species, and at which developmental stage and tissues these genes are expressed, could influence its specificity and sensitivity to odorants^16, 17^.

The role of OBPs in host and mate choice has previously been investigated in several species^5, 18, 19^, since these genes are important targets for natural and sexual selection. Here, we studied differential expression of OBP genes in two closely related species of the *fraterculus* group, *Anastrepha fraterculus* and *Anastrepha obliqua*. These species show important ecological and reproductive differences^20–24^ that could be related with olfactory perception. Though *A*. *fraterculus* has been associated with a wide number of hosts, it prefers several Myrtaceae fruits^20^, being considered one of the main economic pests in South America. *A*. *obliqua*, on the other hand, though an important pest species as well, has been associated with a smaller number of hosts, several of those Anacardiaceae^21^. Olfactory reception also plays an important role in courtship behavior of these species during lek aggregations, which is a common feature for both species. In that case, females may be able to recognize species-specific pheromones and thus avoid interspecific matings^22–24^.

The differential detection of host odors and reproductive partners triggers several behaviors associated with distinct combinations of odorants and may subject genes in the OBP family to strong selective pressures^25^, which can gradually lead to differentiation of the OBP genes repertoire and their expression levels. A previous study analyzed selection pressures in the repertoire of OBP genes from *A*. *fraterculus* and *A*. *obliqua*, identifying some genes under positive selection^26^. In the present study, we investigated the patterns of expression by qPCR of eight OBP genes in male and female heads of *A*. *fraterculus* and *A*. *obliqua* that showed either evidence of positive selection^26, 27^ or were found to have differential expression between these species. We analyzed how these genes are expressed at different reproductive stages in each species, which could suggest whether these OBPs are more probably involved with food or mate location. Furthermore, we also investigated differential expression of each OBP gene between species, which could indicate genes potentially involved in their differentiation^28^. Since differences in OBP expression patterns may effect different olfactory responses and, consequently, elicit important ecological and behavioral consequences, a better understanding of the expression of these genes may bring important information on the diversification and speciation of closely related species such as those from the *Anastrepha fraterculus* group, which could help develop potential targets for species-specific control of these, and other, pest species.

## Results and Discussion

### Screening of differentially expressed OBP genes between *A*. *fraterculus* and *A*. *obliqua* from RNA-seq data

**Figure 1:**
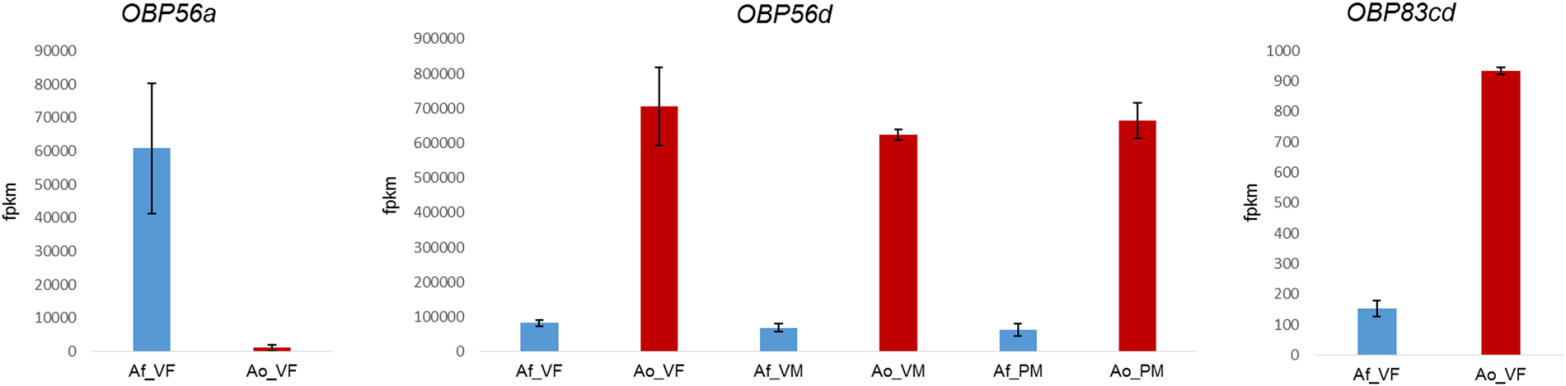
Differentially expressed OBP genes according to RNA-seq data analysis. Af = *A*. *fraterculus* (profiles in blue); Ao = *A*. *obliqua* (profiles in red); VF = virgin female; VM = virgin male; PM = post-mating male. RNA-seq expression was measured by fragments per kilobase of transcript per million mapped reads (fpkm).

Studies in *Drosophila* indicate that the detection of differing host odors by sibling species may be reflected in the expression level of the olfactory genes involved^29, 30^. Therefore, we expected to find some OBP genes to show differential expression between *A*. *fraterculus* and *A*. *obliqua* because, even though they may use common hosts, they have different host preferences and also because individuals seem to be able to recognize pheromones of their own species^22–24, 31^, which are two important ecological and reproductive attributes that may be involved in the species’ differentiation. We looked initially for differentially expressed OBP genes between these two species using RNA-seq data of head tissues^27^ and found three OBP genes significantly differentially expressed (FDR < 0.001): *OBP56a*, *OBP56d* and *OBP83cd* in virgin females and *OBP56d* in virgin and post-mating males (Fig. 1; Table 1). Whereas *OBP56d* and *OBP83cd* were up-regulated in *A*. *obliqua*, *OBP56a* was up-regulated in *A*. *fraterculus*, at a level of differential expression much higher than the other significant gene comparisons here identified (Table 1), which indicate species differences in patterns of expression.

**Table 1:** Significance and fold-change values of the RNA-seq differential expression analyses between *A*. *fraterculus* and *A*. *obliqua* OBP genes.

Gene	Differentially expressed profile	FDR	Up-regulated species	Fold-change
*OBP56a*	VF	8.87E-11	*A*. *fraterculus*	51.89
*OBP56d*	VF	9.55E-32	*A*. *obliqua*	9.10
	VM	6.31E-26		9.03
	PM	1.62E-15		10.68
*OBP83cd*	VF	6.32E-06	*A*. *obliqua*	6.44

VF = virgin female; VM = virgin male; PM = post-mating male. FDR = false discovery rate.

When we contrast the differences in expression between head tissues of *A*. *fraterculus* and *A*. *obliqua* with a study that investigated differences in expression of OBP genes in antennae of three *Drosophila* species which found 19 OBPs differentially expressed between *D*. *sechellia* and *D*. *simulans*, 21 OBPs between *D*. *sechellia* and *D*. *melanogaster* and 20 between *D*. *simulans* and *D*. *melanogaster* ^30^, we notice many fewer differences in gene expression between *A*. *fraterculus* and *A*. *obliqua* than among the *Drosophila* species studied, in which 27 of the 52 members of OBP family were expressed in the antenna^32^, even though their divergence time is comparable. It is possible that there are biological aspects that explain this difference in number of differentially expressed OBPs, but we should mention that these studies used very different methodologies, so part of the difference may be due to the smaller bias of microarray analyses and qPCR used for *Drosophila* when compared to analyses using next-generation transcriptomes, used for *Anastrepha*, despite the use of two library replicates and a deep sequencing coverage for these libraries.

Since some studies in flies have reported that the most relevant differences in gene expression would occur between reproductive stages (instead of different tissues or life stages)^33–35^, we expanded our investigation to include different stages in reproduction. We investigated immature individuals, collected 24 hours after eclosion, to contrast against virgin individuals that were sexually mature, collected 10 days after eclosion, when males and females start showing mating behavior. Furthermore, comparisons between virgin and mated *D*. *melanogaster* females showed that the peak of differentially expressed genes was at 1–3 hours post-mating in the adult^34^, 6 hours post-mating in reproductive tissues^36^ and 72 hours post-mating in head and brain tissues, with the majority of genes (including *OBP99b*) showing lower expression at this later time^33^. Since there was no previous information available for *Anastrepha* species, we considered these results in *Drosophila* to sample post-mating males and females of *Anastrepha* at 3, 6, 12, 24 and 48 hours post-mating. We compared differential expression of the different samples to virgin mature individuals, since we were particularly interested in the effect of sexual maturation and mating. We evaluated expression in seven reproductive profiles (immature and mature individuals and five post-mating profiles) of males and females for *OBP56a*, *OBP56d* and *OBP83cd* by qPCR analyses. Besides, we also used qPCR to compare the patterns of expression of five other OBP genes previously described as evolving under positive selection in *Anastrepha* species: *OBP50a*, *OBP56h*-*1*, *OBP56h*-*2*, *OBP57c* and *OBP99c* ^26, 27^.

### Intraspecific differential expression analysis

We used qPCR to investigate how the eight selected OBP genes were expressed along different reproductive stages in each species, to address two distinct questions: 1) whether immature and mature virgin individuals had significant differential expression and 2) whether expression significantly changes after mating, comparing individually five post-mating profiles against mature virgin individuals. We conducted and reported our species-specific analyses separately for males and females because studies in *A*. *obliqua*, *B*. *dorsalis* and *D*. *melanogaster* showed sex-dependent differences in levels of expression of chemosensory genes, which indicates that they experience, interact with, and adapt to their chemical environments differently^35, 37, 38^. Even though the reference genes here used in real time PCR analyses were originally described for different biological conditions, and only for *A*. *obliqua* ^37^, they were also effective for studies on circadian clock genes of *A*. *fraterculus* ^39^. Our tests indicate that these reference genes are also adequate for the conditions and tissues here tested, since we failed to observe significant differential expression levels among the profiles and species analyzed (data not shown). Since a reliable set of reference genes is critical for real time PCR analyses, these results expand once again the conditions, species, and tissues for which these reference genes are suitable.

**Figure 2:**
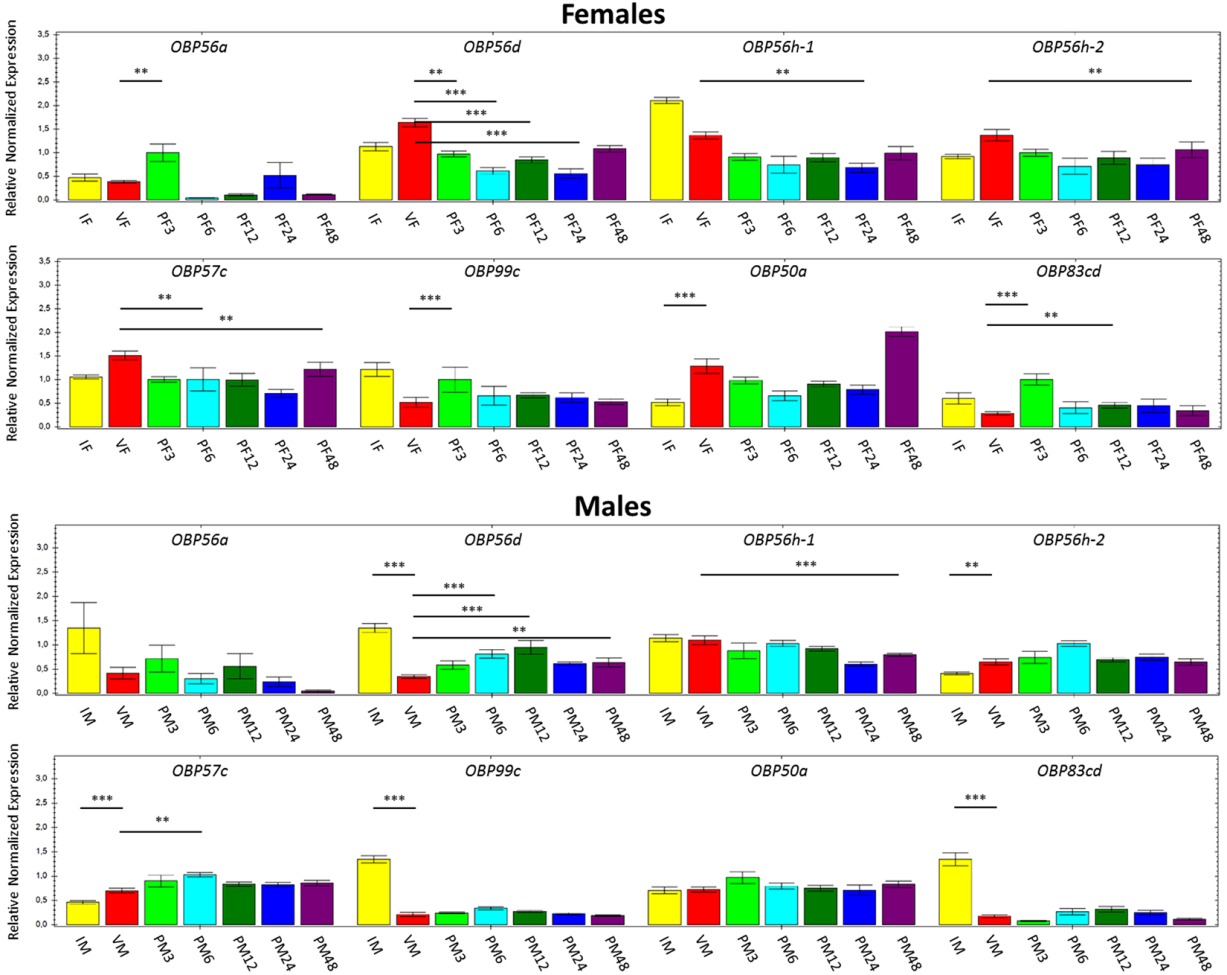
Relative normalized expression of OBP genes in *A*. *fraterculus* reproductive stages according to qPCR analyses. IF = immature female; VF = virgin female; PF3 = 3 h post-mating female; PF6 = 6 h post-mating female; PF12 = 12 h post-mating female; PF24 = 24 h post-mating female; PF48 = 48 h post-mating female; IM = immature male; VM = virgin male; PM3 = 3 h post-mating male; PM6 = 6 h post-mating male; PM12 = 12 h post-mating male; PM24 = 24 h post-mating male; PM48 = 48 h post-mating male. Pairs of profiles significantly differentially expressed are show at the endpoints of each line above the bars. Unpaired t-test: **p-value < 0.01; ***p-value < 0.001.

**Figure 3:**
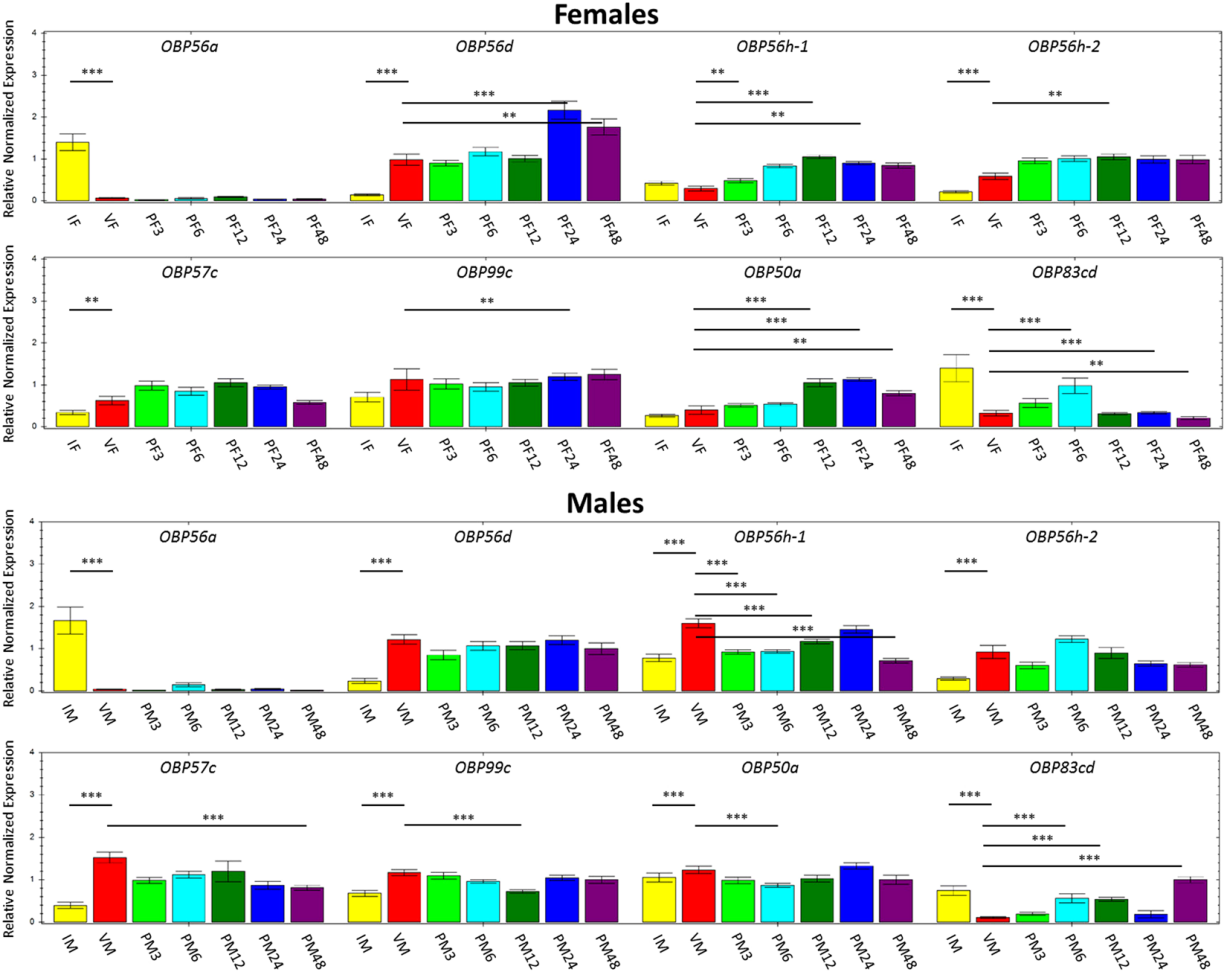
Relative normalized expression of OBP genes in *A*. *obliqua* reproductive stages according to qPCR analyses. IF = immature female; VF = virgin female; PF3 = 3 h post-mating female; PF6 = 6 h post-mating female; PF12 = 12 h post-mating female; PF24 = 24 h post-mating female; PF48 = 48 h post-mating female; IM = immature male; VM = virgin male; PM3 = 3 h post-mating male; PM6 = 6 h post-mating male; PM12 = 12 h post-mating male; PM24 = 24 h post-mating male; PM48 = 48 h post-mating male. Pairs of profiles significantly differentially expressed are show at the endpoints of each line above the bars. Unpaired t-test: **p-value < 0.01; ***p-value < 0.001.

We will refer to immature virgin males and females as immature males and females, mature virgin males and females as virgin males and females and post-mating mature males and females as post-mating males and females, to simplify reading. *A*. *fraterculus* showed significant differential expression for one OBP between immature and virgin females (*OBP50a*, up-regulated in virgin females), and for five OBPs between immature and virgin males (*OBP56d*, *OBP99c* and *OBP83cd*, up-regulated in immature males; *OBP56h*-*2* and *OBP57c*, up-regulated in virgin males) (Fig. 2). We also found three OBPs up-regulated in post-mating females, in comparison with virgin females: *OBP56a* and *OBP99c* three hours after mating (PF3) and *OBP83cd* three and 12 hours after mating (PF3 and PF12), while the other five genes were down-regulated in some of the post-mating profiles. In *A*. *fraterculus* post-mating males, two OBPs were up-regulated: *OBP56d* (PM6, PM12 and PM48) and *OBP57c* (PM6), while *OBP56h*-*1* was down-regulated (PM48) in post-mating males (Fig. 2). We highlight the *OBP56d* that showed opposite patterns between sexes, being down-regulated in post-mating females and up-regulated in post-mating males. *A*. *obliqua* showed significant differential expression for five OBPs between immature and virgin females (*OBP56a* and *OBP83cd*, up-regulated in immature females; *OBP56d*, *OBP56h*-*2* and *OBP57c*, up-regulated in virgin females), and for all OBP genes between immature and virgin males (*OBP56a* and *OBP83cd*, up-regulated in immature, and the other genes up-regulated in virgins) (Fig. 3). These results indicated a similar expression pattern in these profiles between *A*. *obliqua* males and females, which was not found for *A*. *fraterculus*. Furthermore, in *A*. *obliqua* females, six genes were up-regulated in post-mating females, in comparison with virgins: *OBP56d* (PF24 and PF48), *OBP56h*-*1* (PF12 and PF24), *OBP56h*-*2* (PF12), *OBP99c* (PF24), *OBP50a* (PF12, PF24 and PF48) and *OBP83cd* (PF6, PF24 and PF48), whereas in males most OBPs that showed differential expression among the profiles tested were down-regulated in post mating, with the exception of *OBP83cd* (PM6, PM12 and PM48), which was up-regulated (Fig. 3).

Considering the set of OBP genes we tested here, *A*. *obliqua* showed more OBP genes differentially expressed between immature and virgins than *A*. *fraterculus* and, in both species, males had a higher number of OBPs differentially expressed upon maturation (Afra_F = 1; Afra_M = 5; Aobl_F = 5; Aobl_M = 8), maybe due to an earlier reproductive maturation^40^. Before maturation, females tend to search for protein sources and plant hosts for foraging and eventual oviposition, shifting from a post-eclosion developmental phase during which they reach reproductive maturity to one in which they prepare for maximum reproduction^36^. Females seek carbohydrates and proteins for their own development and for that of their eggs. Likewise, immature males spend a large proportion of their time feeding to accumulate energy, which will define their reproductive success^41^. Based on these needs, we hypothesize that genes up-regulated in immature flies are more probably involved with the location of food resources during foraging activity, instead of location of mate partners. Besides, sexual maturation in insects is accompanied by the need to locate a mate and, subsequently, in the case of females, to switch from mate searching to oviposition behavior^7, 42^. In *C*. *capitata*, for instance, studies revealed that sexually mature virgin females are attracted to the odor of male-produced pheromone, while mated females are more interested in finding suitable oviposition sites and are therefore more attracted to the odor of host fruits^42–45^. Considering these findings, we suggest that OBP genes up-regulated in virgins, in comparison with immature flies, may be involved in pheromone perception.

Our comparisons between virgin and post-mating profiles revealed that OBP expression levels of the genes we investigated changed significantly after mating. Whereas in *A*. *fraterculus* females most OBPs analyzed were down-regulated post-mating, in *A*. *obliqua* females we found the opposite, the majority of OBPs were up-regulated post-mating, which is the same pattern observed in *D*. *melanogaster* females^38^. After mating, females become less attracted to males, probably due to a lower expression of pheromones stimulated by seminal fluid proteins transferred from the males, tending to become unreceptive to further mates and look for oviposition sites^33, 46–48^. Thus, OBPs up-regulated in post-mating females could be involved in finding oviposition sites, whereas down-regulated OBPs, in pheromone perception. On the other hand, mature males are actively involved in lek formation, in which the recognition of male pheromones is extremely important for male aggregation and for female attraction^43, 49^, which would explain a few up-regulated OBPs in post-mating males. A transcriptome analysis in *C*. *capitata* showed that sexual maturation induces profound expression changes in females and modest variations in males, whereas post-mating changes were modest in females, and consistent both during maturation and post-mating in males^46^, a pattern that was not repeated for the OBP genes we investigated in *Anastrepha*. In both species, we observed different patterns of expression between males and females, as was previously described for *Drosophila* OBPs^38^. Only *OBP56a* showed the same pattern of expression between males and females of *A*. *obliqua*. Sex-specific gene expression differences found in OBPs were still more likely between virgin and post-mating profiles, since each sex has different behavioral and physiological attributes to maximize their reproductive potentials.

Since we used data from whole head tissues, we must consider that the OBPs here studied may have been expressed in other parts of the head, such as the brain or the taste organs^33, 50^ and not in the olfactory tissues, and therefore would not be involved in olfactory processes. However, in *D*. *melanogaster*, homologues of all eight of these OBPs were expressed in the antennae, especially *OBP56d*, *OBP57c* and *OBP99c*, expressed at high levels in both males and females^51, 52^. Some of the OBP genes described here, *OBP56a*, *OBP56d*, *OBP56h*, *OBP57c* and *OBP99c*, were also found differentially expressed at different reproductive stages of *D*. *melanogaster* ^38^ (pairwise amino acid similarities between *Anastrepha* and *D*. *melanogaster*’s OBPs are shown in the Supplementary Table S1). Although the patterns of expression are not similar, the recurrence of the same OBP genes differentially expressed in both species may indicate a similar importance in these flies’ reproductive process. Furthermore, *OBP56a*, *OBP56d* and *OBP56h* genes from *D*. *melanogaster* belong to the same gene cluster^53^, which may indicate they have the same promoters. Nonetheless, as it was observed for *D*. *melanogaster*, the expression patterns of OBP genes from the same gene cluster may differ in *A*. *fraterculus* and *A*. *obliqua*, suggesting that even OBP genes in the same gene cluster may be regulated independently. This independent regulation is supposed to be a necessary requirement for subfunctionalization or neofunctionalization during evolution, when daughter genes of duplication events either allow refinement and/or expansion in perception of the chemical environment, or yet the acquisition of specialized chemosensory functions^38^.

### Interspecific differential expression analysis

**Figure 4:**
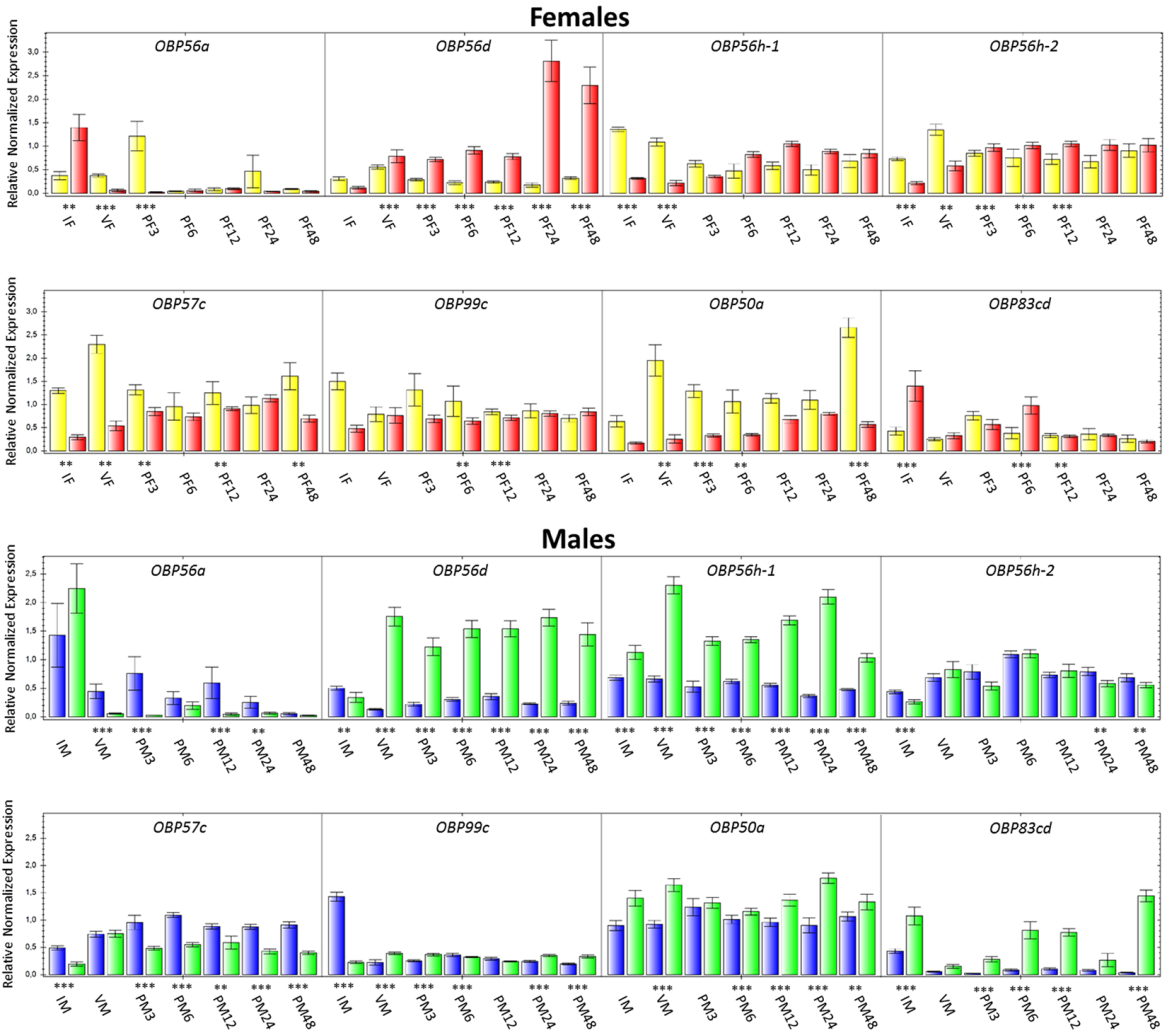
Comparative gene expression of OBP genes between *A*. *fraterculus* and *A*. *obliqua*. Yellow and blue bars represent *A*. *fraterculus* reproductive stages in females and males, respectively. Red and green bars represent *A*. *obliqua* reproductive stages in females and males, respectively. IF = immature female; VF = virgin female; PF3 = 3 h post-mating female; PF6 = 6 h post-mating female; PF12 = 12 h post-mating female; PF24 = 24 h post-mating female; PF48 = 48 h post-mating female. IM = immature male; VM = virgin male; PM3 = 3 h post-mating male; PM6 = 6 h post-mating male; PM12 = 12 h post-mating male; PM24 = 24 h post-mating male; PM48 = 48 h post-mating male. Unpaired t-test: **p-value < 0.01; ***p-value < 0.001.

Differences in OBP gene expression between species could result in different olfactory perception, since it may indicate that species experience, interact with, and adapt to their chemical environments differently^54^. To investigate potential differences in gene expression between *A*. *fraterculus* and *A*. *obliqua*, we investigated expression patterns across several reproductive stages, focusing on the differential expression of individual OBPs. Once again, we restricted our interspecific comparisons of gene expression to the same sex, because OBPs have shown sex-biased gene expression in flies^34, 37, 38^. The comparison between *A*. *fraterculus* and *A*. *obliqua* considering reproductive stages of immature and virgin females (Fig. 4, IF and VF profiles) revealed that most OBPs that displayed differential expression pattern were significantly more expressed in *A*. *fraterculus*, with the exception of *OBP56a* and *OBP83cd* in immature and *OBP56d* in virgins, more expressed in *A*. *obliqua*. This pattern was different in the comparison of males (Fig. 4, IM and VM profiles), since some OBPs (*OBP56a*, *OBP56h*-*2* and *OBP57c*) were more expressed in *A*. *fraterculus*, others (*OBP56h*-*1*, *OBP50a* and *OBP83cd*) were more expressed in *A*. *obliqua*, and yet two of them (*OBP56d* and *OBP99c*) showed an antagonistic pattern, more expressed in immature males of one species but in virgins of the other species.

**Figure 5:**
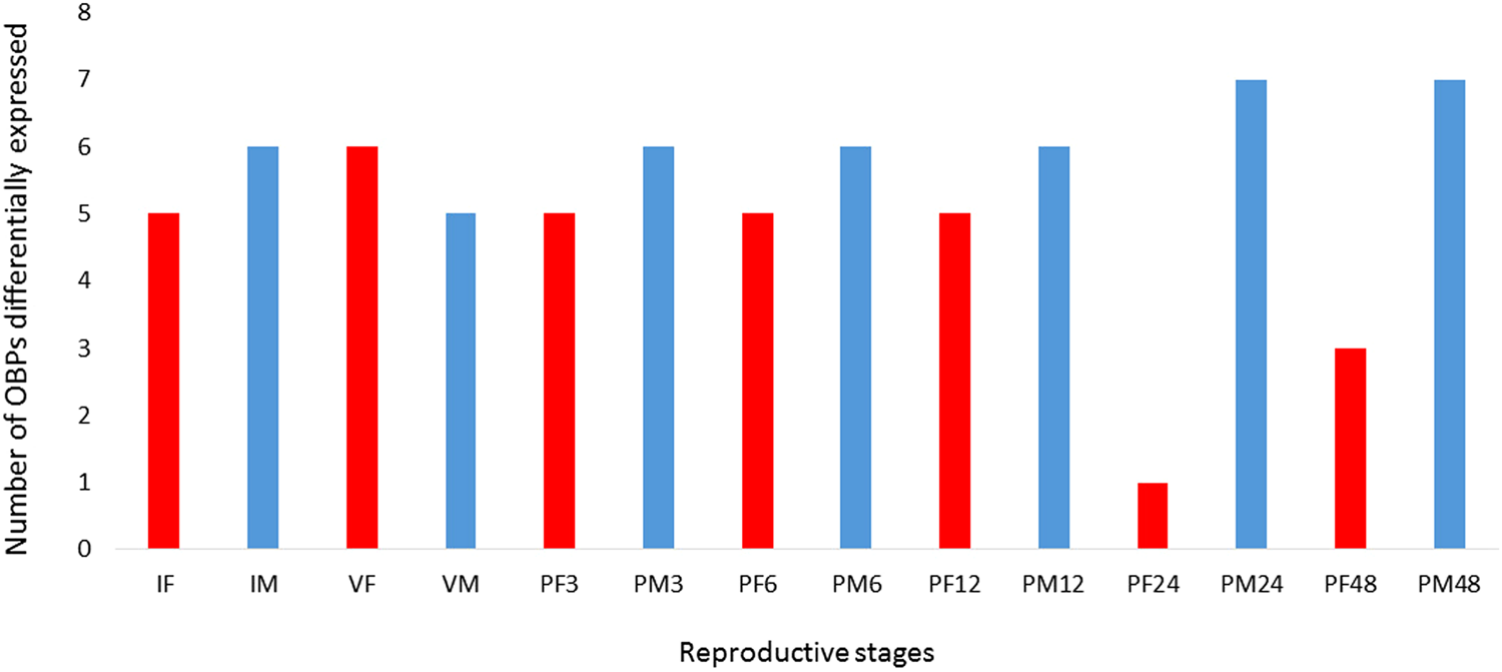
OBPs differentially expressed between *A*. *fraterculus* and *A*. *obliqua* females (red bars) and males (blue bars) for each profile analyzed. IF = immature female; VF = virgin female; PF3 = 3 h post-mating female; PF6 = 6 h post-mating female; PF12 = 12 h post-mating female; PF24 = 24 h post-mating female; PF48 = 48 h post-mating female. IM = immature male; VM = virgin male; PM3 = 3 h post-mating male; PM6 = 6 h post-mating male; PM12 = 12 h post-mating male; PM24 = 24 h post-mating male; PM48 = 48 h post-mating male.

The interspecific comparison among post-mating profiles revealed that *OBP56d*, *OBP56h*-*2* and *OBP57c* showed more profiles significantly differentially expressed between *A*. *fraterculus* and *A*. *obliqua* females (Fig. 4, PF profiles), and *OBP56d*, *OBP56h*-*1*, *OBP57c* and *OBP99c* between males (Fig. 4, PM profiles). The putative orthologs of these genes are very similar between *A*. *fraterculus* and *A*. *obliqua* (See Supplementary Table S2 for pairwise amino acid identities), *OBP56d* being the most divergent, showing 88% similarity across species. We highlight the common occurrence of *OBP56d* and *OBP57c* as differentially expressed in male and female contrasts, and the fact that *OBP56d* had the highest number of differentially expressed profiles between these species, seven profiles for males and six for females. *OBP57c* gene expression was shown to increase in females of *D*. *melanogaster* exposed to odors of other females, which could suggest a function related to female pheromone reception^38^. To the best of our knowledge, no putative functions were associated to *OBP50a*, *OBP56d* and *OBP56h*. Seven OBPs were differentially expressed between males of *A*. *fraterculus* and *A*. *obliqua* at least one day after mating (24 and 48 hours after mating). Interestingly, at the same period, females showed the lowest numbers of OBPs differentially expressed between species (Fig. 5).

Although the qPCR results do not exactly corroborate the RNA-seq expression data, which is not surprising since the profiles are not exactly the same, the comparisons of each OBP expression between *A*. *fraterculus* and *A*. *obliqua* were concordant in both analyses. In summary, all eight OBP genes investigated here by qPCR showed significant differential expression between *A*. *fraterculus* and *A*. *obliqua*, but *OBP56a*, *OBP56d*, *OBP57c* and the two copies of *OBP56h* showed the most divergent patterns between *A*. *fraterculus* and *A*. *obliqua*. The two copies originally described for *OBP56d* for both species were suggested to represent intraspecific variation^26^, so they were considered as a unique gene to conduct the *in silico* gene expression analysis. Although two sequences were also associated with *OBP56d* for *B*. *dorsalis* (81% of pairwise identity between the two copies)^55^, the large pairwise identity between the two copies (94% and 95% for *A*. *fraterculus* and *A*. *obliqua*, respectively) and the single product produced in the qPCR analysis suggests that both sequences are different alleles of a single gene in the *Anastrepha* species studied. The intra- and interspecific variability previously reported, associated with the significant differential expression found between the two species, make *OBP56d* an interesting candidate gene to investigate differentiation among *Anastrepha* species. On the other hand, the two copies of *OBP56h* in *Anastrepha* may have arisen via gene duplication that preceded the divergence of *A*. *fraterculus* and *A*. *obliqua*. These two copies have a pairwise identity of 29% in *A*. *fraterculus* and 33% in *A*. *obliqua* ^26^. The presence of two paralogous copies could have favored functional diversification, and promoted different expression patterns^56^. We clearly observed this differentiation in the interspecific contrast between males, in which *OBP56h*-*1* and *OBP56h*-*2* showed very distinct expression patterns.

Considering that we selected a set of OBP genes for this study that were the most differentiated between *A*. *fraterculus* and *A*. *obliqua*, either because they showed significant differential expression in Illumina transcriptomes, or because they showed significant evidence of positive selection, it is not surprising that all genes here analyzed were differentially expressed between *A*. *fraterculus* and *A*. *obliqua*. Since OBPs are involved in species-specific behaviors related to host and mate location, differences in OBP expression between *A*. *fraterculus* and *A*. *obliqua* could help explain their preference for different host fruits and mate choice. Although we cannot assume that the OBPs here analyzed are in fact responsible for such ecological differences, or would be the sole contributors to these differences, we found significant differences in their expression between *A*. *fraterculus* and *A*. *obliqua*, indicating that these genes are differentially regulated in these species, which may result in different olfactory responses and consequently, lead to important ecological and behavioral consequences. The differences in OBP expression here described may make these genes relevant to investigate diversification and speciation in these and in other species in the *fraterculus* group, as well as be considered targets for species-specific control of these pest species.

## Conclusions

Intraspecific expression analyses at different reproductive stages of eight OBP genes showed that before mating, some OBP genes were more expressed in immature flies (*OBP56a* and *OBP83cd* for *A*. *obliqua*; *OBP56d*, *OBP99c* and *OBP83cd* for *A*. *fraterculus*), whereas others were more expressed in mature virgin flies (*OBP56d*, *OBP56h*-*1*, *OBP56h*-*2* and *OBP57c* for *A*. *obliqua*; *OBP50a*, *OBP56h*-*2* and *OBP57c* for *A*. *fraterculus*). Considering when these genes are expressed, it is possible that the former genes may be more involved in the search for food, whereas the latter genes may be more associated with mating. Furthermore, we found that expression levels of the OBP genes here studied changed significantly after mating in all sexes for both species. Interspecific comparisons showed high levels of differential expression for OBP genes between *A*. *fraterculus* and *A*. *obliqua*, especially *OBP56d*, *OBP57c* and the two copies of *OBP56h*, which suggests that these genes may have played important roles in the evolution and differentiation of these species.

## Methods

### RNA-seq expression analyses

A differential expression analysis was performed between *A*. *fraterculus* and *A*. *obliqua* using transcriptomes of adult heads (accession numbers SRX2030818-SRX2030827 and SRX2030830-SRX2030833)^27^, where the flies’ olfactory organs (antennae and maxillary palps) are located. The data included four profiles per species at different reproductive stages: mature virgin and mature post-mating for both sexes. The cDNA libraries were prepared in duplicates and each consisted of ten pooled individuals. Mature virgin individuals were collected 10 days after eclosion. Post-mating individuals were at least 10 days old and collected 15–20 hours after the first successful mate. More details about the cDNA libraries’ construction and analysis are described elsewhere^27^. We used MAFFT v7 (available online and using default parameters)^57^ to align 22 pairs of orthologous OBPs of *A*. *fraterculus* and *A*. *obliqua* previously identified^26, 37^ (Supplementary Table S2). Based on these alignments, we trimmed each ortholog OBP to obtain sequences with the same length. The set of sequences from each species was used as reference which was used to align that species’ reads and estimate OBP expression. In so doing, we avoided problems in the RNA abundance quantification step arisen by interspecific polymorphism.

Differential expression analyses were conducted separately for each reproductive stage with a methodology that uses statistical tools for transcript quantification, using the scripts: “align_and_estimate_abundance.pl”, “abundance_estimates_to_matrix.pl”, “run_DE_analysis.pl” and “analyze_diff_expr.pl”, included in Trinity package (release 2014-04-13)^58^. We used the parameters –no-mixed –no-discordant –gbar 1000 –end-to-end -k 200 -q -X 800 of the Bowtie 2 software v2.2.4 (release 2014-10-22)^59^ to align the reads from each library back to the respective species reference. The sam files produced by Bowtie 2 were converted to bam files using SAMtools v0.1.19 (release 2013-03-19)^60^. The next step was to estimate transcript abundance by eXpress v1.5.1 (release 2013-08-12)^61^, using the option –no-bias-correct. Finally, differential transcript expression was quantified using edgeR v3.6.8^62^ using TMM (trimmed mean of M-values) scaling normalization that aims to account for differences in RNA across all comparisons between species. Genes with fold-changes ≥4 and a significance p-value ≤ 0.001 for the Fisher exact test, adjusted using the FDR method^63^, were considered as differentially expressed.

### Selected OBP genes and qPCR primers design

We chose a set of OBP genes that showed differential expression in the RNA-seq data analysis, as well as OBP genes that were previously described under positive selection in *A*. *fraterculus* and *A*. *obliqua* ^26, 27^ to perform quantitative PCR (qPCR) analyses. Eight OBP genes were considered, for which two primer pairs were designed, using the software Primer 3 version 4.0.0 (available at http://bioinfo.ut.ee/primer3/). We aligned *Anastrepha* OBP sequences with their putative *D*. *melanogaster*’s orthologs, to identify putative intron positions using MAFFT^57^. Whenever possible, primer pairs were designed across introns to control for potential amplification of genomic DNA. We designed the primers in regions that were not variable between *A*. *fraterculus* and *A*. *obliqua* sequences, which allowed us to use the same primers for both species, although variations still could occur in the amplified fragment. We tested primer concentrations of 0.3, 0.5 and 0.6 *μ*M in a final reaction volume of 10 *μ*l, and used dissociation curve analyses to evaluate primer-specific amplifications for all OBP primer pairs. Once the best concentrations were determined, primer efficiency was tested using eight cDNA concentrations from a serial dilution starting at 5 ng/*μ*l. We used a pool consisting of five heads of *A*. *fraterculus* females, with three technical replicates on these tests. All primers were tested, considering efficiency (E) between 95–105% and standard curve correlation coefficient (R^2^) higher than 0.95^64^. When both primer pairs were efficient, we chose the one with the closest E value to 100% and R^2^ value to 1. Primer pairs selected for qPCR analyses and their results for efficiency tests are shown in the Supplementary Table S3.

### Profiles analyzed by qPCR

Our experimental design for the qPCR analysis consisted of fourteen profiles equally divided between females and males, sampled with three biological replicates, with each replicate composed of a pool of five heads. The profiles analyzed represented three reproductive stages of the adult life, immatures (collected 24 hours after pupae eclosion), mature virgins, referred here simply as virgin individuals (collected 10 days after pupae eclosion) and post-mating (10-days-old individuals after the first successful mating). We collected post-mating samples by mating 10-days-old virgin males and females, maintained at separate cages since eclosion in the same controlled environment room (25 °C, 60–90% humidity and natural photoperiod), in a proportion of 1:1. When mating started, the couple was gently removed to another cage. To avoid incomplete matings, we considered only matings that lasted longer than 40 minutes and captured the very first successful matings for both sexes. After the males had dismounted, females and males were separated and collected at five different times: 3, 6, 12, 24 and 48 hours post-mating. Post-mating individuals were maintained under the same controlled environment until sacrifice, to reduce interferences on their normal gene expression levels.

### Isolation of total RNA and cDNA synthesis

Total RNA was extracted using the TRIzol/chloroform protocol^65^. RNA quality was visualized in agarose gel electrophoresis for integrity and absorbance was measured in NanoVue*™* Plus Spectrophotometer (GE Helthcare). Samples were quantified with Qubit^®^ 2.0 Fluorometer, using the RNA BR assay kit (Invitrogen*™*). Before transcription, total RNA was treated with DNase I Amplification Grade (Invitrogen*™*), according to the manufacturer’s protocol, to remove residual genomic DNA. One *μ*g of treated RNA was converted into cDNA using iScript*™* cDNA Synthesis Kit (Bio-Rad) and cDNA samples were diluted to 5 ng/*μ*l for the qPCR assays.

### qPCR assays

We performed the qPCRs in a CFX96 Touch*™* Real-Time PCR Detection Systems (Bio-Rad), using SsoFast*™* EvaGreen^®^ Supermix (Bio-Rad). Reactions were made with a final primer concentration of 0.6 *μ*M in a final volume of 10 *μ*l, which was the best primer concentration in all primer concentrations tests. Cycling parameters for all primers were 30 seconds at 95 °C, followed by 40 cycles of dissociation at 95 °C for 10 seconds and annealing and extension at 60 °C for 1 minute. A fluorescence reading was made at the end of each extension step. For melt curve analysis we used a protocol with temperatures that varied from 65 °C to 95 °C with increments of 0.5 °C for 5 seconds and continuous fluorescent measurements. No template controls (NTC) and inter-run calibrators were included in all qPCR plates. To check for reproducibility, three technical replicates were carried out for each sample. Relative quantification was calculated for each OBP gene with the Bio-Rad CFX Manager*™* software (Bio-Rad), using the comparative ${2}^{{\rm{\Delta }}{\rm{\Delta }}{C}_{t}}$ method^66^ in contrast with three *Anastrepha* reference genes: *rpS17* (Ribosomal proteinS17), *rpL18* (Ribosomal proteinL18) and *ef1a* (Elongation factor-1*α*)^37^. In these analyses, we contrasted both, the within species differential expression of OBP genes among different reproductive stages, and the between species differential expression of each OBP gene. Data were statistically analyzed by ANOVA followed by Tukey’s test using Prism 5.01 software (GraphPad Software, San Diego, CA, USA). A value of p < 0.01 was considered statistically significant.

## Electronic supplementary material

Supplementary Information
