# Supplementary material for: Odorant-binding proteins expression patterns in recently diverged species of Anastrepha fruit flies
Source: Sci Rep. 2017 May 19;7:2194. doi: 10.1038/s41598-017-02371-2 (PMC5438349; doi:10.1038/s41598-017-02371-2)
Supplement: Supplementary file 1 — Supplementary Information [file 41598_2017_2371_MOESM1_ESM.pdf]

Title: Odorant-binding proteins expression patterns in recently diverged species of *Anastrepha* fruit flies

Emeline Boni Campanini[a*]
E-mail: emelinebc@gmail.com

Carlos Congrains[a]
E-mail: carloscongrains@gmail.com

Felipe Rafael Torres[a]
E-mail: feliperafaeltorres@hotmail.com

Reinaldo Alves de Brito[a]
E-mail: brito@power.ufscar.br

[a] Departamento de Genética e Evolução, Universidade Federal de São Carlos
Rodovia Washington Luís km235 – 13565-905 – São Carlos – São Paulo – Brasil

Supplementary Information

Supplementary Table S1. Pairwise amino acid similarities between *Drosophila melanogaster* OBPs and their *Anastrepha fraterculus* and *A. obliqua* putative orthologs.

Supplementary Table S2. *A. fraterculus* and *A. obliqua* OBP sequences used as reference for the RNA-seq gene expression analyses.

Supplementary Table S3. Primer pairs selected for qPCR analyses and results for efficiency tests.

Supplementary Table S1. Pairwise amino acid similarities between *Drosophila melanogaster* OBPs and their *Anastrepha fraterculus* and *A. obliqua* putative orthologs.

***D. melanogaster* OBP (accession number)**	***Anastrepha* OBP**	**Pairwise amino acid similarity (%) *A. fraterculus***	**Pairwise amino acid similarity (%) *A. obliqua***
OBP56a (NP_611442.1)	*OBP56a*	31.42	36.97
OBP56d (NP_611444.2)	*OBP56d*	58.33	60.14
OBP56h (NP_611448.2)	*OBP56h_1*	64.28	64.28
	OBP56h_2	54.35	52.17
OBP57c (NP_611481.1)	*OBP57c*	42.93	44.26
OBP99c (NP_651711.1)	*OBP99c*	75.16	74.51
OBP50a (NP_725385.1)	*OBP50a*	34.76	31.27
OBP83cd (NP_649612.1)	*OBP83cd*	64.37	63.56

Supplementary Table S2. *A. fraterculus* and *A. obliqua* OBP sequences used as reference for the RNA-seq gene expression analyses.

***A. fraterculus* gene**	**Accession number**	***A. obliqua* gene**	**Accession number**	**Pairwise amino acid identity (%)**
AfraOBP8a	KU317957	*AoblOBP8a*	KU317933	97.42
AfraOBP19a	KU317958	*AoblOBP19a*	KU317934	100
AfraOBP19b	KU317976	*AoblOBP19b*	KU317935	97.67
AfraOBP19c	KU317959	*AoblOBP19c*	KU317936	93.94
AfraOBP19d	KU317960	*AoblOBP19d*	KU317937	99.36
AfraOBP47b	KU317961	*AoblOBP47b*	KU317938	96.37
AfraOBP49a	KU317962	*AoblOBP49a-1*	KU317939	95.79
AfraOBP50a-1	KU317977	*AoblOBP50a*	KU317941	92.75
AfraOBP50e	KU317963	*AoblOBP50e*	KU317942	99.13
AfraOBP56a	KP939314	*AoblOBP56a*	KX018812	95.87
AfraOBP56d-1	KU317979	*AoblOBP56d-1*	KU317943	88.46
AfraOBP56h-1	KU317965	*AoblOBP56h-1*	KU317945	93.55
AfraOBP56h-2	KU317966	*AoblOBP56h-2*	KU317946	96.30
AfraOBP57c	KU317967	*AoblOBP57c*	KU317947	95.48
AfraOBP59a	KU317968	*AoblOBP59a*	KU317948	99.02
AfraOBP83cd	KU317969	*AoblOBP83cd*	KU317949	97.92
AfraOBP83ef	KU317970	*AoblOBP83ef*	KU317950	99.61
AfraOBP83g	KU317971	*AoblOBP83g*	KU317951	95.07
AfraOBP99a	KU317972	*AoblOBP99a*	KU317952	100
AfraOBP99b	KU317973	*AoblOBP99b*	KU317953	98.03
AfraOBP99c	KU317974	*AoblOBP99c*	KU317954	94.67
AfraOBP99d	KU317975	*AoblOBP99d-1*	KU317955	97.35

Supplementary Table S3. Primer pairs selected for qPCR analyses and results for efficiency tests.

Gene	Positive selection	*In silico* differential expression	Sequences accession number	Primers	Sequences	E (%)	R^2
OBP50a	yes[26]	no	Af = KU317977<br>Ao = KU317941	Foward<br>Reverse	CATAGCATCGACGAAAACGG<br>CCGCCTTCTCTAATTGCTCG	97.5	0.997
OBP56a	no	females	Af = KP939314<br>Ao = KX018812	Foward<br>Reverse	GAGGCCAAATTCGATGTGTT<br>ACACTTTTCCCGATCGTTTG	101.6	0.990
OBP56d	no	females and males	Af = KU317979<br>Ao = KU317943	Foward<br>Reverse	AAGTCGTTAGGTCCCTTGG<br>TAGCACTTGTACCTCTCGAG	98.1	0.998
OBP56h-1	yes[26]	no	Af = KU317965<br>Ao = KU317945	Foward<br>Reverse	TGTCACCGTTCTAATGTGTCA<br>ATTATCCTTGGCATCGCTGG	98.5	0.991
OBP56h-2	yes[26]	no	Af = KU317966<br>Ao = KU317946	Foward<br>Reverse	CAATGCGCCATGGTCAAAC<br>CGCTAGCCAACTTTGCATC	102.6	0.995
OBP57c	yes[26]	no	Af = KU317967<br>Ao = KU317947	Foward<br>Reverse	GGCTTGTCTAGAGCAGCACA<br>TTCTCGTCCATGAAGCCCAG	99.1	0.991
OBP83cd	no	females	Af = KU317969<br>Ao = KU317949	Foward<br>Reverse	GTGCGTTACAAGGAGTGGTC<br>TCGCAATTATCCGCCAATGG	101	0.973
OBP99c	yes[27]	no	Af = KU317974<br>Ao = KU317954	Foward<br>Reverse	TGAAGCTGAGGTATTGGCCAT<br>AGACACTTGTGACCGCGATA	99.4	0.998

Af = *A. fraterculus*; Ao = *A. obliqua*; E = efficiency; R^2 = standard curve correlation coefficient.
